# Review of Anton A. Niekerk and Loretta M. Kopelman (eds.) *Ethics and AIDS in Africa: The Challenge to our Thinking*

**DOI:** 10.1186/1742-6405-3-24

**Published:** 2006-10-05

**Authors:** Stuart Rennie

**Affiliations:** 1 Departments of Dental Ecology and Social Medicine, University of North Carolina at Chapel Hill, Chapel Hill, NC 27599-7450, USA

Ethics, AIDS, and Africa – three concepts that conjure up a host of powerful associations. Ethics: our fragile human attempts to negotiate acceptable paths through conflicts of value. AIDS: the world's deadliest epidemic since the Black Plague of the 14^th ^century. And Africa: cradle of humankind, burdened by colonization, famine, poverty and civil war. In what ways do ethics, AIDS and Africa go together, and how is our thinking challenged by their relationship?

Editors Van Niekerk and Kopelman have assembled an impressive list of well-respected authors to deal with this question. Their contributions focus on the impact of AIDS in sub-Saharan Africa and take on some of the key ethical issues raised by HIV/AIDS research, policy and clinical practice in the region. Although rich in details, it is fair to say that four general themes dominate this book: the ethical role of national governments in tackling the AIDS epidemic, with special focus on the South African case; the epidemic and responses to it as reflections of global inequity; the ethical responsibilities of pharmaceutical companies in the struggle against HIV/AIDS, and the dilemmas involved in HIV prevention research, particularly in vaccine studies.

For a book on ethics, there is an unusual amount of agreement. None of the authors seriously doubts the terrifying social impact HIV/AIDS is currently having in sub-Saharan Africa. There is general condemnation of the lack of leadership and intransigence of the South African government in its approach to both rolling out AIDS treatment and redoubling its HIV prevention efforts. There is also no question among the contributors that poverty, lack of education, gender inequality and inadequate health care infrastructure both fuel the spread of the virus and limit access to treatment, and that tackling the epidemic in Africa is inseparable from larger economic, social, political, human rights and development issues.

But there are also interesting cracks in the consensus. Solomon Benatar argues that the AIDS epidemic in Africa has exposed our world as fundamentally inequitable and unstable, and little progress will be made unless current international relations in politics and trade are rethought and remade. Anton Van Niekerk counters that efforts in low-resource countries should focus on what is doable in the short-term, rather than a quixotic pursuit for global reform, and he argues that national governments in Africa cannot be mere passive beneficiaries of international funding – they have an ethical duty to create policies and programs that sustain global AIDS initiatives. David Resnik claims that pharmaceutical companies have ethical responsibilities towards those suffering from AIDS in the developing world, but given the money-making imperatives of big pharma, this responsibility can only realistically take the form of some investment in R&D in the developing world, discounts on drug prices or drug give-aways. For Richard Ashcroft and Udo Schuklenk, such 'charity' on the part of multinational corporations is simply not good enough. When a country is experiencing a devastating public health crisis, compulsory licensing of essential AIDS treatment may be the most effective and ethically justified way of getting medication to those who need it. In this way, Ashcroft and Schuklenk provide the activist slogan "Patients before patents" with a deeper philosophical justification.

Kevin De Cock, WHO's Director of the Department of HIV/AIDS, recently stated at the last International AIDS Conference in Toronto that 'we cannot treat our way out of this epidemic.' HIV prevention research and policies are crucial: the former to find novel ways of preventing new infections, and the latter to channel prevention strategies into practice. The chapters by Keymanthri Moodley, Godfrey Tangwa and Melissa Stobie et. al. offer an excellent overview of the ethical and cultural issues encountered in conducting HIV vaccine research in sub-Saharan Africa with adults and children. Both Tangwa and Moodley tantalizingly claim that the informed consent process – cornerstone of the ethical conduct of biomedical research in the West – inappropriately assumes that the African research participant can be separated from his kinship and community ties. What would count as a more 'culturally sensitive' consent process, however, remains elusive. Stobie et. al. usefully discuss the tensions between ethical concerns for individuals and concern for social groups in HIV vaccine research. On the one hand, it would be in the best interest of children as a class for there to be an efficacious HIV vaccine, at least for children growing up in a high HIV prevalence setting. But it may or may not be in an individual child's best interest to participate in an HIV vaccine trial. The discussion of the deep ambiguities in South African laws and guidelines concerning the best interests of children, the limits of parental consent to research involving children and the determination of risk in pediatric studies is both illuminating and sobering. It is not as if laws and guidance on pediatric research are much clearer elsewhere.

There are some minor limitations to this book. Although the title refers to Africa, the focus is really on sub-Saharan Africa, with a special emphasis on South Africa. To a certain extent, this is understandable and justified. South Africa has an estimated 5.5 million persons living with HIV and approximately 1000 persons dying of AIDS daily. The South Africa government is a reliable source of bizarre HIV-related publicity, be it the President's embrace of rogue scientists who deny HIV causes AIDS, a health minister who suggests garlic and nutritional supplements are as effective as anti-retrovirals in controlling AIDS, or a previous head of the South African AIDS Council who claims a post-coital shower (after sex with an HIV positive woman) can protect him against the virus. South Africa also, as it turns out, has exceptional centers for bioethics and research ethics at Cape Town and Pretoria. Nevertheless, the book might have profited by looking farther afield and incorporating voices of West and East Africans, and Africans living above the Sahara.

Many of the pieces in the book seem to have been originally written around 2001, and therefore the factual data and the ethical commentary based on the data sometimes appear a bit dated. The results of the efforts by the Global Fund, PEPFAR, the Clinton Foundation and the Bill and Melinda Gates Foundation over the last five years are not well-represented. HIV prevention research is currently focused on more on female-controlled methods (microbicides) and controversial surgical interventions (male circumcision) as the development of an effective vaccine still seems a lot way off. Keeping the ethics up to date is clearly a difficult task, given the rapid evolution in disease dynamics and in the political, economic and scientific responses to the HIV/AIDS epidemic.

These minor reservations aside, *Ethics and AIDS in Africa *is a highly illuminating, stimulating and informative guide to the ethical dilemmas faced by researchers, policy-makers and health care providers in the countries hardest hit by HIV/AIDS. The book clearly delivers on its promise that HIV/AIDS, as it continues to ravage low-resource countries of Africa, challenges everyone's ethical thinking.

